# Association of plasma F2-isoprostanes and isofurans concentrations with erythropoiesis-stimulating agent resistance in maintenance hemodialysis patients

**DOI:** 10.1186/s12882-015-0074-9

**Published:** 2015-06-05

**Authors:** Matthew B. Rivara, T. Alp Ikizler, Charles D. Ellis, Rajnish Mehrotra, Jonathan Himmelfarb

**Affiliations:** Division of Nephrology, Department of Medicine, University of Washington, Box 359606, 325 9th Ave., Seattle, WA 98104 USA; Kidney Research Institute, Seattle, WA USA; Division of Nephrology, Department of Medicine, Vanderbilt University Medical Center, Nashville, TN USA; Vanderbilt Center for Kidney Disease, Vanderbilt University Medical Center, Nashville, TN USA

**Keywords:** Chronic inflammation, Erythropoietin, End stage renal disease, Hemodialysis, Oxidative stress, F2-isoprostanes, Isofurans, CRP, IL-6

## Abstract

**Background:**

In patients undergoing maintenance hemodialysis (HD), hyporesponsiveness to erythropoiesis stimulating agents (ESAs) is associated with adverse clinical outcomes. Systemic inflammation is highly prevalent in HD patients and is associated with ESA hyporesponsiveness. Oxidative stress is also highly prevalent in HD patients, but no previous study has determined its association with ESA response. This study assessed the association of plasma markers of oxidative stress and inflammation with ESA resistance in patients undergoing maintenance HD.

**Methods:**

We analyzed data from 165 patients enrolled in the Provision of Antioxidant Therapy in Hemodialysis study, a randomized controlled trial evaluating antioxidant therapy in prevalent HD patients. Linear and mixed-effects regression were used to assess the association of baseline and time-averaged high sensitivity F2-isoprostanes, isofurans, C-reactive protein (hsCRP), and interleukin-6 (IL-6) with ESA resistance index (ERI), defined as the weekly weight-adjusted ESA dose divided by blood hemoglobin level. Unadjusted models as well as models adjusted for potential confounders were examined. Predicted changes in ERI per month over study follow-up among baseline biomarker quartiles were also assessed.

**Results:**

Patients with time-averaged isofurans in the highest quartile had higher adjusted mean ERI compared with patients in the lowest quartile (β = 14.9 ng/ml; 95 % CI 7.70, 22.2; reference group <0.26 ng/ml). The highest quartiles of hsCRP and IL-6 were also associated with higher adjusted mean ERI (β = 10.8 mg/l; 95 % CI 3.52, 18.1 for hsCRP; β = 10.2 pg/ml; 95 % CI 2.98, 17.5 for IL-6). No significant association of F2-isoprostanes concentrations with ERI was observed. Analyses restricted to baseline exposures and ERI showed similar results. Baseline hsCRP, IL-6, and isofurans concentrations in the highest quartiles were associated with greater predicted change in ERI over study follow-up compared to the lowest quartiles (*P* = 0.008, *P* = 0.004, and *P* = 0.04, respectively). There was no association between baseline F2-isoprostanes quartile and change in ERI.

**Conclusions:**

In conclusion, higher concentrations of isofurans, hsCRP and IL-6, but not F2-isoprostanes, were associated with greater resistance to ESAs in prevalent HD patients. Further research is needed to test whether interventions that successfully decrease oxidative stress and inflammation in patients undergoing maintenance HD improve ESA responsiveness.

**Electronic supplementary material:**

The online version of this article (doi:10.1186/s12882-015-0074-9) contains supplementary material, which is available to authorized users.

## Background

Anemia leading to treatment with erythropoiesis-stimulating agents (ESAs) is highly prevalent among patients with end stage renal disease (ESRD) and both the severity of anemia and variability in hemoglobin have been associated with poor clinical outcomes in patients on maintenance dialysis [1–4]. Hyporesponsiveness to ESAs – manifested as an increase in the ESA dose requirement to maintain a constant blood hemoglobin level, a drop in blood hemoglobin on a constant ESA dose, or a failure to achieve a target hemoglobin level on very high doses of ESAs – is also associated with increased risk of adverse outcomes [5–7]. Furthermore, ESA therapy is costly, which is further amplified in patients with ESA hyporesponsiveness [8].

The etiology of ESA resistance in patients undergoing maintenance hemodialysis (HD) is complex and incompletely understood. One likely contributor is chronic systemic inflammation, which has been found to be present in up to 80 % of HD patients [9, 10]. Several prior studies have identified an association between ESA resistance and blood levels of systemic inflammatory markers such as C-reactive protein and interleukin-6 (IL-6) [11–13]. Furthermore, small-scale investigations of anti-inflammatory interventions have suggested a potential benefit on improving ESA responsiveness [14, 15].

Growing evidence suggests that an exaggerated oxidative stress burden may also play a role in inducing ESA resistance in patients with ESRD [16]. Plasma F2-isoprostanes, an established measure of oxidative stress, has been found to be up to four times higher in patients undergoing maintenance HD compared with healthy subjects [17]. Isofurans, a more recently described biomarker of oxidative stress, has been linked to increased risk for cardiovascular disease and kidney dysfunction [18, 19]. Oxidative stress may promote ESA resistance by increasing red blood cell fragility, decreasing red cell life span, and/or reducing endogenous erythropoietin synthesis [20, 21]. No prior study has examined the association of plasma F2-isoprostanes or isofurans with ESA resistance in HD patients. Given the poor clinical outcomes associated with ESA hyporesponsiveness, there is a compelling need to investigate oxidative stress as a novel pathway leading to resistance to ESAs.

Prior studies have shown that oxidative stress and inflammation are synergistically linked. During an inflammatory response, stimulated leukocytes release superoxide and myeloperoxidase, catalyzing oxidative injury and in turn leading to further release of pro-inflammatory cytokines and acute phase proteins through activation of the transcription nuclear factor κB [22]. However, whether oxidative stress and inflammation may act independently to promote ESA hyporesponsiveness in patients with ESRD remains unknown. The primary objective of this study was to test the hypothesis that higher concentrations of inflammatory and oxidative stress biomarkers are each independent associate with greater resistance to ESAs in patients undergoing maintenance HD.

## Methods

### Study population

Patients were identified from the Provision of Antioxidant Therapy in Hemodialysis (PATH) study, a randomized clinical trial comparing the effect of combination antioxidant therapy with mixed tocopherols plus α-Lipoic acid (ALA) versus matched placebo on biomarkers of inflammation and oxidative stress in patients undergoing maintenance dialysis [23]. Inclusion criteria for the PATH study included ESRD receiving thrice-weekly maintenance HD for at least 120 days, age >18 years, and life expectancy >1 year. Exclusion criteria included AIDS; active malignancy; recent or anticipated kidney transplant; hospitalization over the past 30 days; vitamin E, vitamin C, or anti-inflammatory medications over the past 30 days; and history of poor adherence to hemodialysis. Of the 353 participants randomized in the PATH trial, we identified the 165 individuals with completed baseline measurements of F2-isoprostanes, isofurans, high-sensitivity C-reactive protein (hsCRP), interleukin-6 (IL-6), and who were treated with an ESA at all study visits, for inclusion in the present study. All identified patients completed the study. Baseline patient characteristics for participants included in the present study compared to those excluded are presented in Additional file 1: Table S1. The PATH trial was approved by the Western and Vanderbilt University Medical Center institutional review boards, and all patients provided written informed consent before study enrollment.

### Exposures

Patients underwent study visits at baseline and then monthly for 6 months. Baseline assessment included collection of demographic characteristics, body mass index (BMI), comorbid conditions, etiology of ESRD, type of vascular access, and blood for routine chemistries. In addition, blood was obtained at baseline and at month 6 for measurement of biomarkers of inflammation and oxidative stress, which represented the exposures of interest. Oxidative stress was quantified by simultaneous measurement of plasma F_2_-isoprostane and isofurans by gas chromatography–mass spectrometry analysis [24]. Inflammation was quantified by measurement of hsCRP using ELISA (Diagnostic Systems Laboratories, Webster, TX) and IL-6 cytokine concentrations also using ELISA (BioSource International, Camarillo, CA).

### Outcomes

Patients underwent blood collection for measurement of blood hemoglobin at baseline, and at each monthly follow-up visit. In addition, at each study visit, each patient’s last ESA dose and average hemodialysis frequency over the prior month was ascertained. The primary outcome was the ESA resistance index (ERI), calculated as the weekly weight-adjusted ESA dose (units per kilogram of body weight per week) divided by the hemoglobin level (grams per deciliter). For each patient, the ERI was calculated at baseline, and at each monthly follow-up visit.

### Statistical analyses

Baseline data were summarized, stratified by quartile of ERI. Data are presented as mean ± standard deviation, medians with interquartile ranges, and proportions as appropriate. Data were complete for all baseline covariates. Scatter plots were created and Pearson’s correlation coefficients calculated to summarize the relationship among the primary exposure inflammatory and oxidative stress biomarkers. For both the oxidative stress markers (F2-isoprostanes and isofurans) and the inflammatory markers (hsCRP and IL-6), baseline measurements of exposure variables were grouped into quartiles. Time-averaged exposures were generated by taking the mean of the baseline and 6-month follow-up measurements, and were also grouped into quartiles for analysis. Two main analyses were performed. First, multivariable linear regression was performed to determine the relationship of quartiles of baseline F2-isoprostanes, isofurans, hsCRP, and IL-6 with baseline ERI. Referent categories for comparisons were the lowest quartile of each baseline exposure variable: F2-isoprostanes of <0.049 ng/ml, isofurans of <0.25 ng/ml, hsCRP of <19.8 mg/l, and IL-6 of <8.3 pg/ml. Second, the relationship between quartiles of time-averaged oxidative stress and inflammatory markers with mean ERI over 6-month follow-up was assessed using a multilevel mixed-effects linear regression model with unstructured covariance accounting for correlation among repeated measures within a patient. Referent categories for comparisons were the lowest quartile of each time-averaged exposure variable: F2-isoprostanes of <0.049 ng/ml, isofurans of <0.26 ng/ml, hsCRP of <19.8 mg/l, and IL-6 of <8.3 pg/ml.

Linear and mixed effects regressions using biomarker quartiles as the exposure were selected for the main analysis after model diagnostics were applied to regressions using exposures as continuous variables and substantial violations of key model assumptions were identified. Specifically, plots of residuals versus fitted values were examined and revealed both curvature and unequal variances for all four exposures. Additionally, plots of sorted residuals versus theoretical quantiles of the normal distribution revealed evidence of non-normality for all four exposures. Outliers were also identified, but deletion diagnostics did not reveal substantially influential observations. To address these concerns, log transformations of exposures and ERI were performed, and regressions repeated using robust standard errors. These changes did not satisfactorily address the violation of linearity. Thus, for the primary analyses, regression using exposure quartiles was performed to account for non-linearity.

For each primary analysis, three models were examined: (1) unadjusted; (2) adjusted for potential demographic and laboratory confounders including for age, sex, race, BMI, etiology of ESRD, diabetes status, transferrin saturation (serum iron/total iron binding capacity, single-pool Kt/V, and serum parathyroid hormone); and (3) adjusted for model 2 covariates plus log-transformed hsCRP (for analyses using oxidative stress markers as the exposures), or log-transformed isofurans (for analyses using inflammatory markers as the exposures). Additionally, for analyses using mixed-effects linear models, study visit number and randomly assigned treatment group were added as covariates. Adjusted restricted cubic splines with four degrees of freedom were constructed to illustrate the relationships between baseline measures of oxidative stress and inflammation with baseline ERI. Covariates included in the adjusted spline models were the same as for regression model 3. For illustrative purposes, splines and 95 % confidence intervals were superimposed on scatter plots of baseline biomarker values (x-axis) and ERI (y-axis), where each dot represents an individual patient. Three patients with outlying values for IL-6 > 100 pg/ml were excluded only for the purposes of illustrative spline construction. For spline figures, a logarithmic scale was used on the x-axis given the right-skewed distribution of oxidative stress and inflammatory markers.

A secondary analysis was performed to determine the association of quartiles of baseline markers of inflammation and oxidative stress with predicted change in ERI over the 6-month study follow-up. This analysis was conducted using a generalized estimating equation model including interaction terms for exposure quartile and study visit, and was conducted on cohort of 253 participants with non-missing baseline oxidative stress and inflammatory markers, including subjects not treated with ESA during at study visit. Adjustment covariates were identical to primary analysis regression model 3. Statistical comparisons were made between quartiles 4 and 1 for each biomarker exposure. Additionally, statistical significance of the linear trend was over all quartiles was determined.

All analyses were performed using Stata, version 11 (StataCorp LP, College Station, TX; www.stata.com).

## Results

### Baseline characteristics

Baseline characteristics of the study cohort, stratified by quartile of baseline ERI are summarized in Table 1. Compared to patients with baseline ERI <5.49, patients with baseline ERI >19.2 were more likely to be African American, less likely to dialyze using an arteriovenous fistula compared with a graft, and had higher prevalence of cardiovascular disease and greater maintenance HD duration. Additionally, patients with higher baseline ERI had lower serum iron and lower transferrin saturation.

**Table 1:** Baseline characteristics of study participants by quartile of ESA resistance index

	ESA resistance index quartile					
Characteristic	Total (*n* = 165)	<5.49 (*n* = 41)	5.49–< 9.67 (n =41)	9.67–< 19.2 (n =41)	> = 19.2 (n =42)	P-value^a^
Age (yr)	58 ± 12	58 ± 11	61 ± 11	58 ± 13	59 ± 13	0.43
Male Sex, n (%)	95 (58)	22 (54)	29 (71)	23 (56)	21 (50)	0.24
Body mass index (kg/m^2^)	33 ± 8	35 ± 7	31 ± 7	34 (9)	32 (11)	0.12
Maintenance hemodialysis duration (mo)	35 (21, 66)	30 (21, 60)	29 (18, 49)	36 (22, 58)	48 (28, 87)	0.30
Ever smoker, n (%)	94 (57)	20 (49)	29 (71)	24 (59)	21 (50)	0.14
Pre-dialysis systolic blood pressure (mmHg)	152 ± 24	150 ± 23	151 ± 22	154 ± 24	154 ± 27	0.77
Randomized to PATH study treatment, n (%)	78 (47)	20 (49)	20 (49)	18 (44)	20 (48)	0.97
Race, n (%)						0.18
White	59 (36)	15 (37)	19 (46)	16 (39)	9 (21)	-
African American	102 (62)	25 (61)	22 (54)	23 (56)	32 (76)	-
Asian	1 (1)	1 (2)	0 (0)	2 (5)	0 (0)	-
Other	3 (2)	0 (0)	0 (0)	0 (0)	1 (2)	-
Type of vascular access, n (%)						0.74
Fistula	106 (64)	27 (66)	28 (68)	28 (68)	23 (55)	-
Graft	50 (30)	12 (29)	11 (27)	11 (27)	16 (38)	-
Catheter	9 (6)	2 (5)	2 (5)	2 (5)	3 (7)	-
Cause of ESRD, n (%)						0.83
Diabetes	69 (42)	17 (41)	14 (34)	22 (54)	16 (38)	-
Hypertension	66 (40)	16 (39)	17 (41)	14 (34)	19 (45)	-
Glomerulonephritis	11 (7)	4 (10)	3 (7)	2 (5)	2 (5)	-
Polycystic kidney disease	7 (4)	1 (2)	3 (7)	2 (5)	1 (2)	-
Other	12 (7)	3 (7)	4 (10)	1 (2)	4 (10)	-
Comorbid disease, n (%)						
Diabetes	96 (58)	24 (58)	22 (54)	27 (66)	23 (55)	0.67
History of cardiovascular disease	83 (50)	16 (39)	22 (54)	20 (49)	25 (59)	0.29
Medication use						
ESA dose (units/kg/week)	117 (66, 217)	41 (25, 53)	88 (72, 100)	157 (132, 182)	298 (242, 389)	<0.001
IV iron dose (mg/week)	50 (25, 62.5)	50 (25, 62.5)	50 (0, 75)	62.5 (25, 62.5)	62.5 (25, 100)	0.12
Blood laboratory results at enrollment						
spKt/V	1.4 ± 0.4	1.4 ± 0.5	1.4 ± 0.2	1.4 ± 0.6	1.4 ± 0.3	0.91
Albumin (mg/dl)	3.9 ± 0.3	4.0 ± 0.3	4.1 ± 0.2	4.0 ± 0.3	3.9 ± 0.3	0.01
Hemoglobin (mg/dl)	11.6 ± 1.1	11.9 ± 0.8	11.7 ± 0.9	11.8 ± 1.1	11.1 ± 1.3	0.02
Parathyroid hormone (pg/dl)	331 (192, 520)	410 (234, 613)	326 (196, 430)	264 (185, 457)	388 (189, 649)	0.28
Iron (ug/dl)	69.7 ± 21.5	64.8 ± 22.8	67.2 ± 20.7	55.9 ± 20.9	50.7 ± 17.7	<0.001
Transferrin saturation (%)	24 ± 8.7	25 ± 10	28 ± 8.8	24 ± 7.0	23 ± 7.0	0.002
Ferritin (ng/dl)	512 (375, 767)	512 (420, 835)	608 (353, 778)	464 (345, 652)	604 (358, 759)	0.25

Values for categorical variables given as *n* (%); values for continuous variables given as mean ± SD or median (IQR)

*ESA* Erythropoesis stimulating agent, *IV* intravenous, *hsCRP* high-sensitivity C-reactive protein, *IL-6* Interleukin 6

^a^P-value from test for heterogeneity among groups

**Fig. 1:**
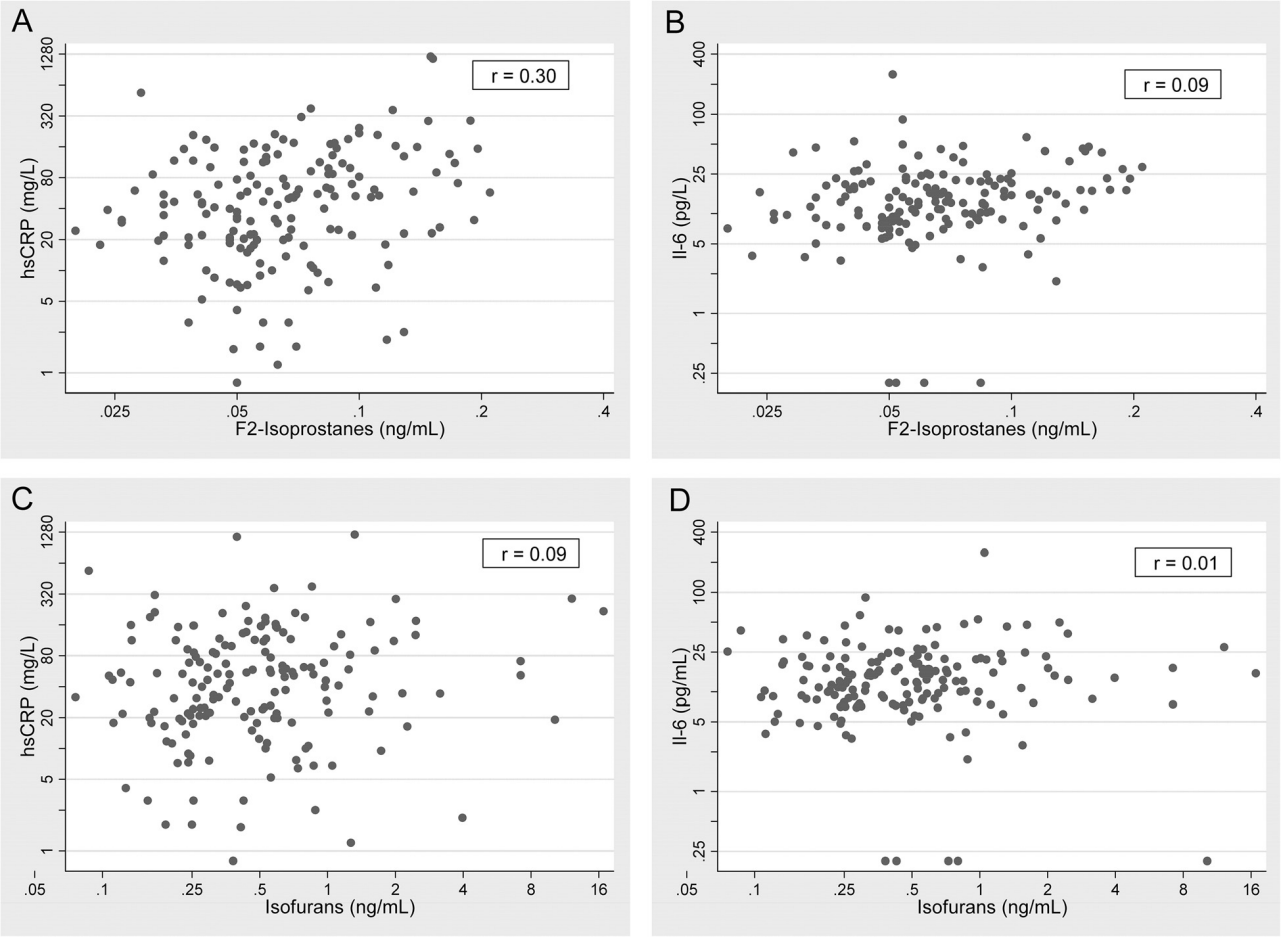
Correlation of baseline F2-isoprostanes and isofurans with baseline hsCRP and IL-6 in maintenance hemodialysis patients. Panels **a** and **b** - F2-isoprostanes and inflammatory markers; Panels **c** and **d** - Isofurans and inflammatory markers

Baseline F2-isoprostane concentrations were positively correlated with hsCRP (*r* = 0.30) (Fig. 1). In contrast, isofurans concentrations were only very weakly correlated with hsCRP (*r* = 0.09), and were not correlated with IL-6 levels (*r* = 0.01). Plots illustrating the relationship of F2-isoprostanes with isofurans and hsCRP with IL-6 are shown in Additional file 1: Figure S1.

### Association of markers of oxidative stress with ERI

**Fig. 2:**
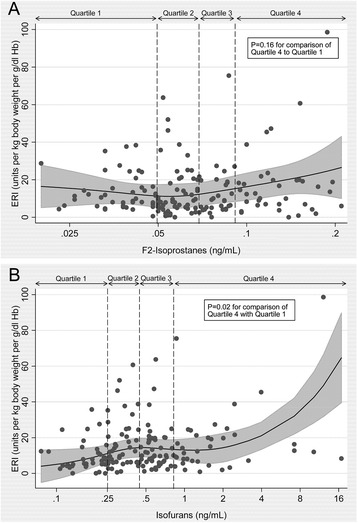
Scatter plots and adjusted restricted cubic splines illustrating relationship of baseline F2-isoprostanes and isofurans with baseline ESA responsiveness index (ERI) in maintenance hemodialysis patients. Panel **a** F2-isoprostanes; **b** Isofurans. Shaded area represents 95 % confidence interval. P-value from multivariable linear regression comparison of quartile 4 to quartile 1, adjustment covariates as per regression model 3

Table 2 shows the associations of baseline F2-isoprostanes and isofurans with baseline ERI. In unadjusted analyses, patients in the highest quartile of isofurans concentrations (>0.71 ng/ml) had significantly higher ERI compared to patients with concentrations <0.25 ng/ml (β = 7.49; 95 % CI 1.14, 13.8; *P* = 0.02). This association persisted following adjustment for demographic variables and transferrin saturation (Model 2), as well as following further adjustment for hsCRP (Model 3). Additionally, patients in the second quartile (0.25–< 0.45 ng/ml) but not the third quartile (0.45–< 0.73 ng/ml) also had significantly higher ERI relative to those in the lowest quartile. This association remained significant across adjustment models, though the significance was attenuated with greater adjustment. In contrast, no significant association between quartile of F2-isoprostanes and ERI was observed, regardless of level of adjustment. Figure 2 illustrates the continuous adjusted relationships between baseline concentrations of F2-isoprostanes and isofurans with ERI.

**Table 2:** Association of quartiles of baseline markers of oxidative stress with baseline ESA resistance index (ERI) in maintenance hemodialysis patients

	Concentration	Model 1^a^			Model 2^b^			Model 3^c^		
		β	95 % CI	P-value^d^	β	95 % CI	P-value^d^	β	95 % CI	P-value^d^
F2-Isoprostanes (ng/ml)	<0.049	Reference	-	-	Reference	-	-	Reference	-	-
	0.049–< 0.063	−0.54	−6.06, 4.98	0.85	−3.02	−9.13, 3.09	0.33	−2.87	−9.09, 3.35	0.36
	0.063–< 0.087	−2.03	−6.10, 2.04	0.33	−2.21	−7.95, 3.52	0.45	−2.40	−7.94, 3.14	0.39
	≥0.087	5.04	−2.00, 12.1	0.16	8.00	−1.53, 17.5	0.10	6.44	−2.54, 15.4	0.16
Isofurans (ng/ml)	<0.25	Reference	-	-	Reference	-	-	Reference	-	-
	0.25–< 0.45	6.67	1.55, 11.8	0.01	5.61	0.24, 10.98	0.04	5.29	0.003, 10.6	0.050
	0.45–< 0.73	4.42	−0.31, 9.25	0.07	3.99	−1.74, 9.74	0.17	2.41	−3.52, 8.34	0.42
	≥0.73	7.49	1.14, 13.8	0.02	9.45	1.49, 17.4	0.02	9.10	1.25, 16.9	0.02

ERI defined as the weekly weight-adjusted ESA dose (units/kg/week) divided by the baseline hemoglobin level (g/dl)

*ESA* Erythropoeisis stimulating agent, *hsCRP* High-sensitivity C-reactive protein

^a^ Unadjusted

^b^ Adjusted for age, sex, race, body mass index, etiology of end stage renal disease, diabetes status, transferrin saturation (serum iron/total iron binding capacity), Kt/V, and serum parathyroid hormone

^c^ Adjusted for Model 2 covariates plus hsCRP

^d^ P-values obtained from comparing regression coefficients to coefficient for lowest quartile of each inflammatory marker

Table 3 shows the associations of time-averaged markers of oxidative stress with mean ESA resistance over follow-up, using linear mixed-effects models. The results of the analyses using time-averaged isofurans as the exposure variable were not substantially different than those using only the baseline measurements, with the second and highest quartiles of isofurans concentration associated with higher mean ERI (reference group: patients with time-averaged isofurans concentration <0.26 ng/ml), regardless of the specific adjustment model. In contrast to the analyses using baseline measurements, patients with time-averaged F2-isoprostanes in the highest quartile had significantly higher mean ERI compared to patients with concentrations <0.049 ng/ml (β = 9.82; 95 % CI 1.78, 17.9; *P* = 0.02) with adjustment for baseline demographic and laboratory variables. However, following full adjustment for potential confounders including for hsCRP (Model 3), this association was no longer significant.

**Table 3:** Association of quartiles of time-averaged markers oxidative stress with mean ESA resistance index (ERI) over 6-month follow-up in maintenance hemodialysis patients

	Concentration	Model 1^a^			Model 2^b^			Model 3^c^		
		β	95 % CI	P-value^d^	β	95 % CI	P-value^d^	β	95 % CI	P-value^d^
F2-Isoprostanes (ng/ml)	<0.049	Reference	-	-	Reference	-	-	Reference	-	-
	0.049–< 0.066	2.78	−3.68, 9.24	0.40	4.65	−2.89, 12.2	0.23	4.61	−2.74, 11.9	0.22
	0.066–< 0.086	4.41	−2.05, 10.9	0.18	7.60	0.01, 15.2	0.050	7.31	−0.10, 14.7	0.05
	≥0.086	6.18	−0.28, 12.6	0.06	9.82	1.78, 17.9	0.02	7.47	−0.58, 15.5	0.07
Isofurans (ng/ml)	<0.26	Reference	-	-	Reference	-	-	Reference	-	-
	0.26–< 0.44	10.46	4.24, 16.7	0.001	8.17	1.29, 15.0	0.02	7.33	0.55, 14.1	0.03
	0.44–< 0.79	5.79	−0.44, 12.0	0.07	5.05	−2.18, 12.3	0.17	5.49	−1.62, 12.6	0.13
	≥0.79	11.5	5.36, 17.7	<0.001	16.4	9.10, 23.7	<0.001	14.9	7.70, 22.2	<0.001

ERI defined as the weekly weight-adjusted ESA dose (units/kg/week) divided by the baseline hemoglobin level (g/dl)

*ESA* Erythropoeisis stimulating agent, *hsCRP* High-sensitivity C-reactive protein

^a^ Unadjusted

^b^ Adjusted for age, sex, race, body mass index, etiology of end stage renal disease, diabetes status, transferrin saturation (serum iron/total iron binding capacity), Kt/V, serum parathyroid hormone, study visit, and treatment assignment

^c^ Adjusted for Model 2 covariates plus hsCRP

^d^ P-values obtained from comparing regression coefficients to coefficient for lowest quartile of each inflammatory marker

Comparing predicted change in ERI per month over six-month study follow-up, patients with baseline isofurans concentrations >0.73 ng/ml had a significantly higher predicted monthly ERI increase compared to patients with isofurans <0.25 n/gml (6.26 ng/ml/month; 95 % CI 0.19, 12.33; *P* = 0.04) (Table 4). In contrast, there was no significant difference among quartiles of baseline F2-isoprostanes concentrations with respect to monthly ERI change. Neither oxidative stress marker was associated with a linear trend in ERI change across quartiles.

**Table 4:** Comparison of adjusted change in ESA resistance index per month over study follow-up among baseline oxidative stress and inflammatory marker quartiles

	Change in ESA Resistance Index per Month^a^					
Marker	Quartile 1	Quartile 2	Quartile 3	Quartile 4	Quartile 4 to 1 Comparison P-value	Overall trend P-value^b^
F2-Isoprostanes (ng/ml/month)	.76 (.07,1.45)	2.54 (−3.64,8.73)	1.65 (−4.62,7.93)	5.95 (−.45,12.35)	0.07	0.63
Isofurans (ng/ml/month)	.65 (−.02,1.32)	6.87 (.83,12.92)	2.41 (−3.74,8.56)	6.26 (.19,12.33)	0.04	0.99
hsCRP (mg/l/month)	.15 (−.53,.84)	2.15 (−3.96,8.27)	4.99 (−1.12,11.09)	8.27 (2.17,14.38)	0.008	0.59
IL-6 (pg/ml/month)	.52 (−.15,1.19)	2.89 (−3.05,8.84)	2.7 (−3.35,8.75)	8.79 (2.83,14.75)	0.004	0.83

^a^ Adjusted for age, sex, race, body mass index, etiology of end stage renal disease, diabetes status, transferrin saturation (serum iron/total iron binding capacity), Kt/V, serum parathyroid hormone, study visit, and treatment assignment

^b^ P-value for trend over biomarker quartiles

### Association of markers of inflammation with ERI

In unadjusted analyses, similar to the observed associations between isofurans and ERI, patients with baseline hsCRP ≥101 mg/l had higher mean baseline ERI compared with patients with hsCRP <19.8 mg/dl (β = 8.45; 95 % CI 1.63, 15.3; *P* = 0.01) (Table 5). Conversely, patients with baseline hsCRP falling into the middle two quartiles did not have significantly higher baseline ERI compared with patients with hsCRP in the lowest quartile. Patients with baseline IL-6 concentrations in the highest quartile of ≥20.9 pg/ml, but not those with measurements in the middle two quartiles, had significantly higher ERI compared to patients with IL-6 < 8.3 pg/ml (β = 9.15; 95 % CI 2.78, 15.5; *P* = 0.005). Upon adjustment for demographic factors and baseline transferrin saturation, and further adjustment for oxidative stress as measured by isofurans concentration, these observed associations remained statistically significant for IL-6, but were only of borderline significance for hsCRP (Table 5).

**Table 5:** Association of quartiles of baseline markers of inflammation with baseline ESA resistance index (ERI) in maintenance hemodialysis patients

	Concentration	Model 1^a^			Model 2^b^			Model 3^c^		
		β	95 % CI	P-value^d^	β	95 % CI	P-value^d^	β	95 % CI	P-value^d^
hsCRP (mg/l)	<19.8	Reference	-	-	Reference	-	-	Reference	-	-
	19.8–<45.7	3.32	−1.44, 8.08	0.17	2.92	−2.42, 8.25	0.28	3.49	−1.98, 8.95	0.21
	45.7–<101	3.34	−1.59, 8.27	0.18	4.98	−1.27, 11.24	0.12	4.93	−1.32, 11.2	0.12
	≥101	8.45	1.63, 15.3	0.02	7.84	−0.38, 16.1	0.06	7.40	−0.04, 14.8	0.05
IL-6 (pg/ml)	<8.3	Reference	-	-	Reference	-	-	Reference	-	-
	8.3–<13.1	2.20	−2.38, 6.78	0.34	2.49	−2.81, 7.80	0.35	3.78	−2.02, 9.57	0.19
	13.1–<20.9	5.17	0.70, 9.64	0.02	2.59	−3.42, 8.59	0.39	2.36	−3.44, 8.16	0.42
	≥20.9	9.15	2.78, 15.5	0.005	8.00	0.52, 15.5	0.04	7.82	0.86, 14.8	0.03

ERI defined as the weekly weight-adjusted ESA dose (units/kg/week) divided by the baseline hemoglobin level (g/dl)

*ESA* erythropoeisis stimulating agent, *hsCRP* high-sensitivity C-reactive protein, *IL-6* Interleukin-6

^a^ Unadjusted

^b^ Adjusted for age, sex, race, body mass index, etiology of end stage renal disease, diabetes status, transferrin saturation (serum iron/total iron binding capacity), Kt/V, and serum parathyroid hormone

^c^ Adjusted for Model 2 covariates plus isofurans

^d^ P-values obtained from comparing regression coefficients to coefficient for lowest quartile of each inflammatory marker

Table 6 presents the results of analyses assessing the association of time-averaged markers of inflammation with mean ERI over follow-up, using linear mixed-effects models. For time-averaged IL-6, the results were substantially similar to the analyses using only baseline measurements, with higher mean ERI observed in patients with IL-6 in the highest quartile, regardless of the level of adjustment. In contrast, for time-averaged hsCRP, patients with marker concentrations in the highest quartile had significantly higher mean ERI not only in the unadjusted model, but also in the fully adjusted model (β = 10.8 mg/l; 95 % CI 3.52, 18.1; *P* = 0.004).

**Table 6:** Association of quartiles of time-averaged markers of inflammation with mean ESA resistance index (ERI) over 6-month follow-up in maintenance hemodialysis patients

	Concentration	Model 1^a^			Model 2^b^			Model 3^c^		
		β	95 % CI	P-value^d^	β	95 % CI	P-value^d^	β	95 % CI	P-value^d^
hsCRP (mg/l)	<22.1	Reference	-	-	Reference	-	-	Reference	-	-
	22.1–<53.4	−1.15	−7.51, 5.21	0.72	3.09	−5.21, 11.4	0.47	4.27	−3.56, 12.1	0.29
	53.4–<117	2.29	−4.07, 8.64	0.48	4.76	−3.26, 12.8	0.25	4.65	−2.89, 12.2	0.23
	≥117	7.53	1.21, 13.8	0.02	11.2	3.41, 18.9	0.005	10.8	3.52, 18.1	0.004
IL-6 (pg/ml)	<9.4	Reference	-	-	Reference	-	-	Reference	-	-
	9.4–<14.1	2.01	−4.26, 8.28	0.53	2.29	−4.96, 9.54	0.54	2.30	−4.54, 9.14	0.51
	14.1–<20.8	6.39	0.11, 12.7	0.05	4.43	−2.88, 11.7	0.24	3.07	−3.86, 10.0	0.39
	≥20.8	10.7	4.42, 16.9	0.001	11.4	3.69, 19.0	0.004	10.2	2.98, 17.5	0.006

ERI defined as the weekly weight-adjusted ESA dose (units/kg/week) divided by the baseline hemoglobin level (g/dl)

*ESA* Erythropoeisis stimulating agent, *hsCRP* High-sensitivity C-reactive protein, *IL-6* Interleukin-6

^a^ Unadjusted

^b^ Adjusted for age, sex, race, body mass index, etiology of end stage renal disease, diabetes status, transferrin saturation (serum iron/total iron binding capacity), Kt/V, serum parathyroid hormone, study visit, and treatment assignment

^c^ Adjusted for Model 2 covariates plus isofurans

^d^ P-values obtained from comparing regression coefficients to coefficient for lowest quartile of each inflammatory marker

**Fig. 3:**
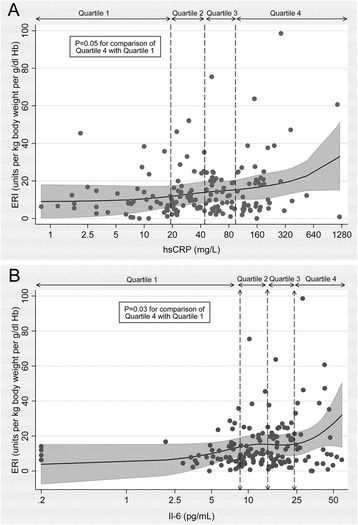
Scatter plots and adjusted restricted cubic splines illustrating relationship of baseline high sensitivity C-reactive protein (hsCRP) and interleukin-6 (IL-6) with baseline ESA responsiveness index (ERI) in maintenance hemodialysis patients. Panel **a** hsCRP; **b** IL-6. Shaded area represents 95 % confidence interval. P-value from multivariable linear regression comparison of quartile 4 to quartile 1, adjustment covariates as per regression model 3

Figure 3 illustrates the continuous unadjusted relationship between hsCRP and IL-6 with ERI. Examination of the restricted cubic splines further supported the use of quantile regression rather than continuous linear regression, as the relationship between inflammatory markers and ERI was curvilinear. Similar to the results of the regression analyses, an association between greater inflammation and higher ERI was evident only at higher concentrations of hsCRP and IL-6.

Comparing predicted change in ERI per month over six-month study follow-up, patients with baseline hsCRP concentrations >101 mg/l had a significantly higher predicted monthly ERI increase compared to patients with hsCRP <19.8 mg/l (8.27 mg/l/month; 95 % CI 2.17, 14.38; *P* = 0.008) (Table 4). Similarly, patients with baseline IL-6 in the highest quartile had significantly greater monthly change in ERI over follow-up compared to patients in the lowest quartile (*P* = 0.004). Neither inflammatory marker was associated with a linear trend in ERI change across quartiles.

### Sensitivity analysis

Two sets of sensitivity analyses were performed. In the first set, the study cohort was expanded to include participants of PATH who had one or more study visits during which they were untreated with ESAs. Inclusion of these additional participants increased the total number of subjects from 165 to 253. Linear regressions assessing the association between baseline biomarker concentrations and baseline ERI, and linear mixed-effects regressions assessing the association between time-averaged exposures and mean ERI were repeated with the expanded cohort. The results of these analyses were similar to those obtained in the primary analysis (Additional file 1: Tables S2–S5). On exception was that patients with baseline IL-6 in the highest quartile had significantly higher ERI compared to those in the lowest quartile only in an unadusted analysis; following full adjustment, this association was no longer presents (Additional file 1: Table S4).

In the second set of sensitivity analyses, only patients who were treated with ESA at each study visit, and who were assigned to the control group in the PATH trial were included in the study cohort. This restricted cohort to 87 participants was then analyzed using linear mixed effects regression to determine the association of time-averaged inflammatory and oxidative stress exposures with ERI. This approach was employed to eliminate the possible impact of antioxidant treatment on the time-averaged exposures. The results of this set of analyses are reported in Additional file 1: Tables S6 and S7. Patients with time-averaged hsCRP in the top two quartiles had significantly greater mean ERI compared to those in the lowest quartile (β = 11.9 mg/l; 95 % CI 2.91, 20.8; *P* = 0.01). There were no significant associations between quartile of IL-6, F2-isoprostanes or isofurans and ERI.

## Discussion

In this study examining the association of markers of inflammation and oxidative stress on ESA resistance in patients undergoing maintenance dialysis, we found that higher concentrations of hsCRP, IL-6 and isofurans, but not F2-isoprostanes, were associated with increased resistance to ESAs, as measured by ERI. These associations were evident in unadjusted models, and also following adjustment for potential demographic and laboratory confounders. Additionally, these associations were not linear, with higher ERI only observed for patients with inflammatory biomarkers in the top quartile, and for patients with isofurans in the second and fourth but not the third quartile. Finally, the association between increased concentrations of isofurans and ERI remained significant following adjustment for hsCRP, and that for the markers of inflammation and ERI remained significant with adjustment for isofurans. This observation suggests possible distinct biological mechanisms leading from inflammation versus oxidative stress to ESA resistance.

Multiple mechanisms may explain how systemic inflammation leads to inhibition of erythropoiesis and thus to resistance to ESAs in patients undergoing maintenance HD. Patient with ESRD have many contributory factors to systemic inflammation, including uremic toxin buildup, dialysate microcontamination, exposure to indwelling catheters and synthetic grafts, and high prevalence of comorbid diabetes and atherosclerotic disease [25]. Inflammation has in turn been shown to lead to activation of macrophages with removal of circulating erythrocytes, down-regulation of erythropoietin receptors, and dysregulated cell signaling following activation of the erythropoietin receptor [26, 27]. Additionally, inflammatory cytokines have been demonstrated to decrease erythropoietin production, suppress proliferation of red cell precursors in the bone marrow, and decrease release of mature erythrocytes into the circulation [28–30].

The data on the contribution of oxidative stress to anemia in patients with ESRD undergoing maintenance dialysis are far more limited. Oxidative stress has been shown to be associated with anemia through downregulation of erythropoietin synthesis, decreased red cell production, and increased erythrocyte fragility. In particular, human erythrocyte nitric oxide synthesis and production of peroxynitrite are activated by oxidative stress leading to upregulation of superoxide dismutase, increased erythrocyte membrane rigidity, and shortening of red cell lifespan [21, 31]. In clinical studies, lipid peroxidation of the erythrocyte cell membrane has been positively associated with markers of red cell destruction, and with resistance to administered ESAs in patients undergoing maintenance HD [20]. Additionally, elevated circulating 8-hydroxy-2’-deoxyguanosine, a product of oxidized DNA, has been associated with greater ESA resistance in HD patients [32]. Furthermore, oxidative stress activates cationic erythrocyte membrane channels permeable to ionic calcium, anionic channels permeable to chloride ions, and apoptotic caspases, all of which may lead to upregulation of programmed erythrocyte cell death [33, 34]. In our study, we found that isofurans were poorly correlated with both hsCRP and IL-6, and there remained a predictive relationship between isofurans and ERI even after adjustment for hsCRP as a biomarker of inflammation. These observations provide evidence that the pathway leading from oxidative stress to ESA resistance is likely distinct from the more well-characterized pathway linking inflammation with ESA resistance. Our findings are in line with those of other studies that have identified pathways leading to ESA resistance that are independent of inflammation. Kalim et al. have recently demonstrated the association of non-enzymatic carbamylation of serum albumin with erythropoietin resistance in ESRD patients, even after adjustment for serum markers of systemic inflammation [35].

In our study, we found an unexpected non-linear relationship between quartiles of isofurans and ESA resistance that was particular notable in the time-averaged analyses. Specifically, we observed higher ERI in patients with isofurans falling not only in the highest quartile, but also in the middle-low quartile. No significant difference was found among patients with isofurans in the third quartile. Certainly one possible explanation for this finding is a statistical one, namely absence of a sufficient sample size to provide adequate power to detect a difference of 5.5 ng/ml in isofurans concentration between the first and third quartiles. Indeed, the β coefficient estimates for the first and third quartiles were 7.3 versus 5.5 ng/ml, numerically quite similar. Alternatively, a true non-linear relationship may exist. Studies of DNA mutagens that act via oxidative stress have shown non-linear dose-effect relationships, with multiple threshold “plateaus” that correspond to the action of cellular defense mechanisms such as reactive oxygen species scavengers and antioxidant enzymes [36]. Similarly, a non-linear relationship between cigarette smoke exposure and cardiovascular risk has also been demonstrated [37]. Thus, in our study, stepping from the second to the third quartile of oxidative stress could theoretically trigger an additional protective mechanism that may reinstate the balance beteween oxidants and antioxidants. In the case of ESA resistance in patients undergoing maintenance HD, such a trigger could partially restore ESA responsiveness. Stepping to the highest level of oxidative stress may overwhelm cellular defense mechanisms leading to ESA resistance. Given that such an explanation remains speculative, further basic and translational research is needed to elucidate the connection between oxidative stress, anemia and ESA resistance.

We observed that high concentrations of isofurans but not F2-isoprostanes were associated with higher ERI. This difference in the predictive role of these two products of free radical-induced peroxidation of arachadonic acid on the degree of ESA resistance was an unexpected finding. Prior studies have shown that relative formation of lipid peroxidation products, including F2-isoprostanes and isofurans, is profoundly influenced by ambient oxygen concentration [38]. In particular, as oxygen concentration increases, F2-isoprostanes formation becomes disfavored, and isofurans formation becomes more favored. In highly oxygenated tissues, such as the central nervous system and the kidneys, isofurans concentrations can exceed F2-isoprostanes levels by greater than two-fold [38]. At the cellular level, elevated levels of reactive oxygen species, as have been demonstrated in ESRD, have been shown to be associated with impaired mitochondrial respiration [39, 40]. Impaired mitochondrial function with blockade of the electron transport chain may then lead to impaired oxygen utilization and buildup of intracellular oxygen molecules with concomitant increased intracellular oxygen concentrations. Independent of blood hemoglobin levels, such increased oxygen concentrations favor isofurans production over F2-isoprostanes production. Furthermore, impaired signaling along the erythropoietin axis is associated with decreased mitochondrial biogenesis, and greater mitochondrial dysfunction, also leading to impaired oxygen utilization, buildup of oxygen tension, and subsequent predominance of isofurans [41–43]. These observations together are one possible explanation for why we observed an association between ERI and isofurans, but not F2-isoprostanes. However, this explanation is theoretical and further research is needed to determine the cellular pathways leading to differential oxidative stress biomarker production.

Our study has multiple strengths. The PATH trial is the largest published trial of antioxidant therapy conducted to date in patients undergoing maintenance HD. All analyzed patients thus had excellent follow-up and were well characterized. Additionally, the lipid peroxidation biomarkers (F2-isoprostanes and isofurans) were analyzed at a reference quality laboratory using gas chromatography/mass spectrometry, thus providing gold standard measures of oxidative stress. Furthermore, repeated measurements of erythropoietin dose response over a 6-month period allowed use of a linear mixed-effects model to incorporate longitudinal data on ESA resistance, increasing the reliability of the observed associations.

Our study also has some limitations. First, though we identified a number of important associations with ESA resistance, it is unclear whether these are causal. Second, the use of data from a clinical trial population may decrease the generalizability of the observed findings. Third, though the results from the PATH trial showed no significant impact of antioxidant treatment status on ESA resistance over study follow-up, it is possible that treatment status may impact the association between inflammatory or oxidative stress markers and ERI. We have attempted to account for this by controlling for trial group assignment in our multivariable regression models, by the possibility of residual confounding remains. Fourth, patients enrolled in the PATH trial were slightly younger and had a higher mean BMI than the general United States ESRD population, which may limit generalizeabilty. Recent work, however, has shown that age does not predict ESA response in either pediatric or adult patients undergoing dialysis [44]. Additionally, though BMI is associated with ESA dose in HD patients, the correlation is weak, and calculation of ERI incorporates body weight [45, 46]. Additionally, we have adjusted for both age and BMI in our final adjustment models. A final limitation is that given the observational nature of our study, there remains the possibility of residual confounding by unmeasured variables on the association between the measured biomarkers inflammation and oxidative stress with ESA resistance.

## Conclusions

In conclusion, we found that in patients undergoing maintenance HD, higher concentrations of isofurans, hsCRP, and IL-6 but not F2-isoprostanes, were associated with increased resistance to erythropoiesis-stimulating agents. The association between isofurans and ERI persisted even after adjustment for levels of inflammation. These findings support that oxidative stress is an important contributor to ESA resistance in the ESRD population, and suggest a pathway independent of systemic inflammation. Future studies should test whether interventions that successfully decrease oxidative stress and inflammation in patients undergoing maintenance dialysis lead to reductions in ESA dose requirements.

## Additional file

Additional file 1: Table S1. Baseline Characteristics of Participants Included Compared to Those Excluded. **Table S2.** Association of quartiles of baseline markers of oxidative stress with baseline ESA resistance index (ERI) in maintenance hemodialysis patients, including subjects not treated with ESA during one or more study visits during follow-up (*n* = 253). **Table S3.** Association of quartiles of time-averaged markers oxidative stress with mean ESA resistance index (ERI) over 6-month follow-up in maintenance hemodialysis patients, including subjects not treated with ESA during one or more study visits during follow-up (*n* = 253). **Table S4.** Association of quartiles of baseline markers of inflammation with baseline ESA resistance index (ERI) in maintenance hemodialysis patients, including subjects not treated with ESA during one or more study visits during follow-up (*n* = 253). **Table S5.** Association of quartiles of time-averaged markers of inflammation with mean ESA resistance index (ERI) over 6-month follow-up in maintenance hemodialysis patients, including subjects not treated with ESA during one or more study visits during follow-up (*n* = 253). **Table S6.** Association of quartiles of time-averaged markers oxidative stress with mean ESA resistance index (ERI) over 6-month follow-up in maintenance hemodialysis patients, excluding subjects in PATH antioxidant treatment arm (*n* = 87). **Table S7.** Association of quartiles of time-averaged markers of inflammation with mean ESA resistance index (ERI) over 6-month follow-up in maintenance hemodialysis patients, excluding subjects in PATH antioxidant treatment arm (*n* = 87). **Figure S1.** Correlation of baseline inflammatory and oxidative stress markers in maintenance hemodialysis patients.

## Abbreviations

ESA: Erythropoiesis-stimulating agent
ERI: Erythropoiesis-stimulating agent resistance index
ESRD: End stage renal disease
HD: Hemodialysis
IL-6: Interleukin-6
hsCRP: High-sensitivity C-reactive protein
BMI: Body mass index
ELISA: Enzyme-linked immunosorbent assay
ALA: α-Lipoic acid
IL-1: Inteleukin-1
IV: Intravenous
