# Supplementary material for: Association of plasma F2-isoprostanes and isofurans concentrations with erythropoiesis-stimulating agent resistance in maintenance hemodialysis patients
Source: BMC Nephrol. 2015 Jun 5;16:79. doi: 10.1186/s12882-015-0074-9 (PMC4455324; doi:10.1186/s12882-015-0074-9)
Supplement: Additional file 1: Table S1. — Baseline Characteristics of Participants Included Compared to Those Excluded. Table S2. Association of quartiles of baseline markers of oxidative stress with baseline ESA resistance index (ERI) in maintenance hemodialysis patients, including subjects not treated with ESA during one or more study visits during follow-up (n = 253). Table S3. Association of quartiles of time-averaged markers oxidative stress with mean ESA resistance index (ERI) over 6-month follow-up in maintenance hemodialysis patients, including subjects not treated with ESA during one or more study visits during follow-up (n = 253). Table S4. Association of quartiles of baseline markers of inflammation with baseline ESA resistance index (ERI) in maintenance hemodialysis patients, including subjects not treated with ESA during one or more study visits during follow-up (n = 253). Table S5. Association of quartiles of time-averaged markers of inflammation with mean ESA resistance index (ERI) over 6-month follow-up in maintenance hemodialysis patients, including subjects not treated with ESA during one or more study visits during follow-up (n = 253). Table S6. Association of quartiles of time-averaged markers oxidative stress with mean ESA resistance index (ERI) over 6-month follow-up in maintenance hemodialysis patients, excluding subjects in PATH antioxidant treatment arm (n = 87). Table S7. Association of quartiles of time-averaged markers of inflammation with mean ESA resistance index (ERI) over 6-month follow-up in maintenance hemodialysis patients, excluding subjects in PATH antioxidant treatment arm (n = 87). Figure S1. Correlation of baseline inflammatory and oxidative stress markers in maintenance hemodialysis patients. [file 12882_2015_74_MOESM1_ESM.docx]

**Table S1: Baseline Characteristics of Participants Included Compared to Those Excluded**

| **Characteristic** | **Included (n = 165)** | **Excluded (n = 188)** |
| --- | --- | --- |
| Age (yr) | 59 ± 12 | 59 ± 13 |
| Male Sex, n (%) | 95 (58) | 103 (58) |
| Body mass index (kg/m^2^) | 33 ± 8 | 30 ± 7 |
| Maintenance hemodialysis duration (mo) | 35 (21, 66) | 32 (19, 61) |
| Ever smoker, n (%) | 94 (57) | 103 (68) |
| Pre-dialysis systolic blood pressure (mmHg) | 152 ± 24 | 150 ± 26 |
| **Race, n (%)** |  |  |
| White | 59 (36) | 78 (44) |
| African American | 102 (62) | 95 (53) |
| Asian | 1 (1) | 1 (1) |
| Other | 3 (2) | 5 (3) |
| **Type of vascular access, n (%)** |  |  |
| Fistula | 106 (64) | 120 (66) |
| Graft | 50 (30) | 51 (28) |
| Catheter | 9 (5) | 10 (6) |
| **Cause of ESRD, n (%)** |  |  |
| Diabetes | 69 (42) | 72 (40) |
| Hypertension | 66 (40) | 68 (38) |
| Glomerulonephritis | 11 (7) | 6 (3) |
| Polycystic kidney disease | 7 (4) | 6 (3) |
| Interstitial nephritis | 0 (0) | 2 (1) |
| Other | 12 (7) | 26 (14) |
| **Comorbid disease, n (%)** |  |  |
| Diabetes | 96 (58) | 96 (53) |
| History of cardiovascular disease | 83 (50) | 87 (48) |
| **Medication use** |  |  |
| **ESA dose (units/kg/week)** | 117 (66, 217) | 69 (0, 212) |
| **IV iron dose (mg/week)** | 50 (25, 62.5) | 50 (15.6, 62.5) |
| **Blood laboratory results at enrollment** |  |  |
| spKt/V | 1.4 ± 0.4 | 1.4 ± 0.5 |
| Albumin (mg/dl) | 4.0 ± 0.3 | 4.0 ± 0.3 |
| Hemoglobin (mg/dl) | 11.6 ± 1.1 |  |
| Parathyroid hormone (pg/dl) | 331 (192, 520) | 318 (198, 470) |
| Iron (ug/dl) | 60 ± 21 | 73 ± 58 |
| Transferrin saturation (%) | 26 ± 9 | 31 ± 23 |
| Ferritin (ng/dl) | 512 (375, 767) | 548 (336, 762) |
| hsCRP (mg/l) | 45.7 (19.8, 101) | 29.5 (15.2, 87.2) |
| IL-6 (pg/ml) | 13.1 (8.3, 21.0) | 12.3 (8.7, 21.2) |
| F2 isoprostane (ng/ml) | 0.063 (0.049, 0.087) | 0.06 (0.04, 0.09) |
| Isofurans (ng/ml) | 0.45 (0.25, 0.73) | 0.39 (0.21, 0.69) |

Values for categorical variables given as *n* (%), whereas values for continuous variables given as mean ±SD or median (IQR) as appropriate.

Abbreviations: ESA, Erythropoesis stimulating agent; hsCRP, high-sensitivity C-reactive protein; IL-6, Interleukin 6; spKt/V: single-pool Kt/V

**Table S2:** Association of quartiles of baseline markers of oxidative stress with baseline ESA resistance index (ERI) in maintenance hemodialysis patients, including subjects not treated with ESA during one or more study visits during follow-up (n=253)

|  | Concentration | Model 1^a^ | | |  | Model 2^b^ | | |  | Model 3^c^ | | |
| --- | --- | --- | --- | --- | --- | --- | --- | --- | --- | --- | --- | --- |
|  |  | β | 95% CI | P-value^d^ |  | β | 95% CI | P-value^d^ |  | β | 95% CI | P-value^d^ |
| **F2-Isoprostanes**  **(ng/ml)** | <0.046 | Reference | - | - |  | Reference | - | - |  | Reference | - | - |
|  | 0.046-< 0.062 | 2.51 | -2.79, 7.80 | 0.35 |  | -2.77 | -8.06, 2.52 | 0.30 |  | -2.06 | -7.36, 3.25 | 0.45 |
|  | 0.062-<0.088 | -0.74 | -5.03, 3.56 | 0.74 |  | -2.26 | -7.94, 3.41 | 0.43 |  | -1.97 | -7.49, 3.56 | 0.48 |
|  | ≥0.088 | 4.33 | -2.26, 10.9 | 0.20 |  | 2.41 | -4.46, 9.28 | 0.49 |  | 1.62 | -4.83, 8.06 | 0.62 |
|  |  |  |  |  |  |  |  |  |  |  |  |  |
| **Isofurans (ng/ml)** | <0.25 | Reference | - | - |  | Reference | - | - |  | Reference | - | - |
|  | 0.25-<0.43 | 6.54 | 1.80, 11.3 | 0.007 |  | 3.92 | -0.43, 8.28 | 0.08 |  | 4.13 | -0.25, 8.51 | 0.06 |
|  | 0.43-<0.71 | 4.82 | 0.67, 8.97 | 0.02 |  | 3.26 | -1.75, 8.27 | 0.20 |  | 2.65 | -2.35, 7.65 | 0.30 |
|  | ≥.71 | 8.35 | 1.93, 14.8 | 0.01 |  | 7.62 | 1.58, 13.7 | 0.01 |  | 6.92 | 1.09, 12.7 | 0.02 |

ERI defined as the weekly weight-adjusted ESA dose (units/kg/week) divided by the baseline hemoglobin level (g/dl). Abbreviations: ESA, erythropoeisis stimulating agent; hsCRP, high-sensitivity C-reactive protein

^a^ Unadjusted

^b^ Adjusted for age, sex, race, body mass index, etiology of end stage renal disease, diabetes status, transferrin saturation (serum iron/total iron binding capacity), Kt/V, and serum parathyroid hormone.

^c^ Adjusted for Model 2 covariates plus hsCRP

^d^ P-values obtained from comparing regression coefficients to coefficient for lowest quartile of each inflammatory marker **Table S3:** Association of quartiles of time-averaged markers oxidative stress with mean ESA resistance index (ERI) over 6-month follow-up in maintenance hemodialysis patients, including subjects not treated with ESA during one or more study visits during follow-up (n=253)

|  | Concentration | Model 1^a^ | | |  | Model 2^b^ | | |  | Model 3^c^ | | |
| --- | --- | --- | --- | --- | --- | --- | --- | --- | --- | --- | --- | --- |
|  |  | β | 95% CI | P-value^d^ |  | β | 95% CI | P-value^d^ |  | β | 95% CI | P-value^d^ |
| **F2-Isoprostanes**  **(ng/ml)** | <0.048 | Reference | - | - |  | Reference | - | - |  | Reference | - | - |
|  | 0.048-<0.064 | 1.91 | -3.82, 7.63 | -.51 |  | 1.95 | -4.19, 8.10 | 0.53 |  | 2.19 | -3.77, 8.16 | 0.47 |
|  | 0.064-<0.087 | 3.12 | -2.69, 8.93 | 0.29 |  | 5.28 | -0.83, 11.4 | 0.09 |  | 5.04 | -0.89, 11.0 | 0.10 |
|  | ≥0.087 | 5.94 | 0.17, 11.7 | 0.04 |  | 5.83 | -0.58, 12.2 | 0.08 |  | 4.55 | -1.71, 10.8 | 0.15 |
|  |  |  |  |  |  |  |  |  |  |  |  |  |
| **Isofurans (ng/ml)** | <0.26 | Reference | - | - |  | Reference | - | - |  | Reference | - | - |
|  | 0.26-<0.44 | 6.74 | 1.11, 12.38 | 0.02 |  | 5.76 | 0.15, 11.4 | 0.04 |  | 5.13 | -0.34, 10.6 | 0.07 |
|  | 0.44-<0.77 | 2.30 | -3.34, 7.94 | 0.42 |  | 2.45 | -3.40, 8.30 | 0.41 |  | 2.27 | -3.43, 7.97 | 0.44 |
|  | ≥0.77 | 8.40 | 2.78, 14.0 | 0.003 |  | 11.1 | 5.18, 17.0 | <0.001 |  | 9.82 | 4.01, 15.6 | 0.001 |

ERI defined as the weekly weight-adjusted ESA dose (units/kg/week) divided by the baseline hemoglobin level (g/dl). Abbreviations: ESA, erythropoeisis stimulating agent; hsCRP, high-sensitivity C-reactive protein

^a^ Unadjusted

^b^ Adjusted for age, sex, race, body mass index, etiology of end stage renal disease, diabetes status, transferrin saturation (serum iron/total iron binding capacity), Kt/V, serum parathyroid hormone, study visit, and treatment assignment

^c^ Adjusted for Model 2 covariates plus hsCRP

^d^ P-values obtained from comparing regression coefficients to coefficient for lowest quartile of each inflammatory marker

**Table S4:** Association of quartiles of baseline markers of inflammation with baseline ESA resistance index (ERI) in maintenance hemodialysis patients, including subjects not treated with ESA during one or more study visits during follow-up (n=253)

|  | Concentration | Model 1^a^ | | |  | Model 2^b^ | | |  | Model 3^c^ | | |
| --- | --- | --- | --- | --- | --- | --- | --- | --- | --- | --- | --- | --- |
|  |  | β | 95% CI | P-value^d^ |  | β | 95% CI | P-value^d^ |  | β | 95% CI | P-value^d^ |
| **hsCRP (mg/l)** | <17.9 | Reference | - | - |  | Reference | - | - |  | Reference | - | - |
|  | 17.9 – <38.6 | 0.34 | -4.18, 4.89 | 0.56 |  | 3.18 | -1.39, 7.77 | 0.17 |  | 2.99 | -1.57, 7.54 | 0.20 |
|  | 38.6 – <96.2 | 3.11 | -1.53, 7.74 | 0.19 |  | 6.67 | 1.60, 11.7 | 0.01 |  | 6.40 | 1.21, 11.6 | 0.02 |
|  | ≥96.2 | 9.19 | 2.12, 16.3 | 0.01 |  | 8.45 | 1.63, 15.3 | 0.02 |  | 7.93 | 1.68, 14.2 | 0.01 |
|  | <17.9 |  |  |  |  |  |  |  |  |  |  |  |
| **IL-6 (pg/ml)** | 17.9 – <38.6 | Reference | - | - |  | Reference | - | - |  | Reference | - | - |
|  | 38.6 – <96.2 | 1.20 | -2.81, 5.22 | 0.56 |  | 1.31 | -3.17, 5.78 | 0.57 |  | 1.81 | -2.91, 6.54 | 0.45 |
|  | ≥96.2 | 2.55 | -1.40, 6.50 | 0.21 |  | -1.06 | -5.87, 3.75 | 0.66 |  | -1.50 | -6.27, 3.27 | 0.54 |
|  | <17.9 | 9.24 | 2.43, 12.1 | 0.008 |  | 3.68 | -2.31, 9.67 | 0.23 |  | 3.26 | -2.38, 8.89 | 0.26 |

ERI defined as the weekly weight-adjusted ESA dose (units/kg/week) divided by the baseline hemoglobin level (g/dl). Abbreviations: ESA, erythropoeisis stimulating agent; hsCRP, high-sensitivity C-reactive protein; IL-6, Interleukin-6

^a^ Unadjusted

^b^ Adjusted for age, sex, race, body mass index, etiology of end stage renal disease, diabetes status, transferrin saturation (serum iron/total iron binding capacity), Kt/V, and serum parathyroid hormone.

^c^ Adjusted for Model 2 covariates plus isofurans

^d^ P-values obtained from comparing regression coefficients to coefficient for lowest quartile of each inflammatory marker**Table S5:** Association of quartiles of time-averaged markers of inflammation with mean ESA resistance index (ERI) over 6-month follow-up in maintenance hemodialysis patients, including subjects not treated with ESA during one or more study visits during follow-up (n=253)

|  | Concentration | Model 1^a^ | | |  | Model 2^b^ | | |  | Model 3^c^ | | |
| --- | --- | --- | --- | --- | --- | --- | --- | --- | --- | --- | --- | --- |
|  |  | β | 95% CI | P-value^d^ |  | β | 95% CI | P-value^d^ |  | β | 95% CI | P-value^d^ |
| **hsCRP (mg/l)** | <20.9 | Reference | - | - |  | Reference | - | - |  | Reference | - | - |
|  | 20.9 - <45.7 | -0.58 | -6.17, 5.02 | 0.84 |  | 2.48 | -3.64, 8.60 | 0.43 |  | 3.30 | -2.67, 9.28 | 0.28 |
|  | 45.7 - <115 | 1.49 | -4.11, 7.07 | 0.60 |  | 4.57 | -1.48, 10.6 | 0.14 |  | 4.33 | -1.56, 10.2 | 0.15 |
|  | ≥115 | 9.17 | 3.60, 14.7 | 0.001 |  | 10.6 | 4.65, 16.6 | <0.001 |  | 10.3 | 4.54, 16.1 | <0.001 |
|  |  |  |  |  |  |  |  |  |  |  |  |  |
| **IL-6 (pg/ml)** | <9.4 | Reference | - | - |  | Reference | - | - |  | Reference | - | - |
|  | 9.4 - <14.3 | 2.39 | -3.23, 8.02 | 0.40 |  | 3.10 | -2.33, 8.53 | 0.26 |  | 3.03 | -2.26, 8.33 | 0.26 |
|  | 14.3 - <21.8 | 5.76 | 0.13, 11.4 | 0.04 |  | 6.28 | 0.40, 12.2 | 0.04 |  | 4.61 | -1.22, 10.4 | 0.12 |
|  | ≥21.8 | 9.32 | 3.72. 14.9 | 0.001 |  | 7.63 | 1.80, 13.5 | 0.01 |  | 7.55 | 1.86, 13.2 | 0.01 |

ERI defined as the weekly weight-adjusted ESA dose (units/kg/week) divided by the baseline hemoglobin level (g/dl). Abbreviations: ESA, erythropoeisis stimulating agent; hsCRP, high-sensitivity C-reactive protein; IL-6, Interleukin-6

^a^ Unadjusted

^b^ Adjusted for age, sex, race, body mass index, etiology of end stage renal disease, diabetes status, transferrin saturation (serum iron/total iron binding capacity), Kt/V, serum parathyroid hormone, study visit, and treatment assignment

^c^ Adjusted for Model 2 covariates plus isofurans

^d^ P-values obtained from comparing regression coefficients to coefficient for lowest quartile of each inflammatory marker

**Table S6:** Association of quartiles of time-averaged markers oxidative stress with mean ESA resistance index (ERI) over 6-month follow-up in maintenance hemodialysis patients, excluding subjects in PATH antioxidant treatment arm (n=87)

|  | Concentration | Model 1^a^ | | |  | Model 2^b^ | | |  | Model 3^c^ | | |
| --- | --- | --- | --- | --- | --- | --- | --- | --- | --- | --- | --- | --- |
|  |  | β | 95% CI | P-value^d^ |  | β | 95% CI | P-value^d^ |  | β | 95% CI | P-value^d^ |
| **F2-Isoprostanes**  **(ng/ml)** | <0.048 | Reference | - | - |  | Reference | - | - |  | Reference | - | - |
|  | 0.048-<0.064 | 2.41 | -4.78, 9.59 | 0.51 |  | 3.93 | -4.10, 12.0 | 0.34 |  | 3.64 | -4.40, 11.7 | 0.38 |
|  | 0.064-<0.087 | 5.13 | -2.13, 12.4 | 0.17 |  | 8.65 | 0.58, 16.7 | 0.04 |  | 8.26 | 0.18, 16.4 | 0.05 |
|  | ≥0.087 | 1.50 | -5.77, 8.76 | 0.69 |  | 3.83 | -4.94, 12.6 | 0.39 |  | 2.57 | -6.49, 11.6 | 0.58 |
|  |  |  |  |  |  |  |  |  |  |  |  |  |
| **Isofurans (ng/ml)** | <0.26 | Reference | - | - |  | Reference | - | - |  | Reference | - | - |
|  | 0.26-<0.44 | 5.29 | -1.89, 12.5 | 0.15 |  | 3.51 | -4.05, 11.1 | 0.36 |  | 4.29 | -3.29. 11.9 | 0.27 |
|  | 0.44-<0.77 | 2.61 | -4.56, 9.79 | 0.48 |  | 2.62 | -6.15, 11.4 | 0.56 |  | 4.39 | -4.70, 13.5 | 0.34 |
|  | ≥0.77 | 2.58 | -4.60, 9.76 | 0.48 |  | 1.23 | -7.38, 9.84 | 0.78 |  | 0.47 | -8.15, 9.09 | 0.91 |

ERI defined as the weekly weight-adjusted ESA dose (units/kg/week) divided by the baseline hemoglobin level (g/dl). Abbreviations: ESA, erythropoeisis stimulating agent; hsCRP, high-sensitivity C-reactive protein

^a^ Unadjusted

^b^ Adjusted for age, sex, race, body mass index, etiology of end stage renal disease, diabetes status, transferrin saturation (serum iron/total iron binding capacity), Kt/V, serum parathyroid hormone, and study visit

^c^ Adjusted for Model 2 covariates plus hsCRP

^d^ P-values obtained from comparing regression coefficients to coefficient for lowest quartile of each inflammatory marker

**Table S7:** Association of quartiles of time-averaged markers of inflammation with mean ESA resistance index (ERI) over 6-month follow-up in maintenance hemodialysis patients, excluding subjects in PATH antioxidant treatment arm (n=87)

|  | Concentration | Model 1^a^ | | |  | Model 2^b^ | | |  | Model 3^c^ | | |
| --- | --- | --- | --- | --- | --- | --- | --- | --- | --- | --- | --- | --- |
|  |  | β | 95% CI | P-value^d^ |  | β | 95% CI | P-value^d^ |  | β | 95% CI | P-value^d^ |
| **hsCRP (mg/l)** | <20.9 | Reference | - | - |  | Reference | - | - |  | Reference | - | - |
|  | 20.9 - <45.7 | 0.82 | -6.40, 8.04 | 0.82 |  | 4.87 | -2.99, 12.7 | 0.23 |  | 5.01 | -2.99, 13.0 | 0.22 |
|  | 45.7 - <115 | 0.54 | -6.69, 7.76 | 0.88 |  | 4.34 | -3.70, 12.4 | 0.29 |  | 4.31 | -3.82, 12.4 | 0.30 |
|  | ≥115 | 3.08 | -4.14, 10.3 | 0.40 |  | 11.8 | 2.96, 20.7 | 0.01 |  | 11.9 | 2.91, 20.8 | 0.01 |
|  |  |  |  |  |  |  |  |  |  |  |  |  |
| **IL-6 (pg/ml)** | <9.4 | Reference | - | - |  | Reference | - | - |  | Reference | - | - |
|  | 9.4 - <14.3 | 1.23 | -5.93, 8.39 | 0.74 |  | 1.74 | -5.35, 8.82 | 0.63 |  | 1.77 | -5.58, 9.12 | 0.64 |
|  | 14.3 - <21.8 | 5.08 | -2.09, 12.2 | 0.17 |  | 5.92 | -1.65, 13.5 | 0.13 |  | 5.92 | -1.73, 13.6 | 0.13 |
|  | ≥21.8 | 3.64 | -3.52, 10.8 | 0.32 |  | -1.68 | -9.83, 6.47 | 0.69 |  | -1.67 | -9.94, 6.60 | 0.69 |

ERI defined as the weekly weight-adjusted ESA dose (units/kg/week) divided by the baseline hemoglobin level (g/dl). Abbreviations: ESA, erythropoeisis stimulating agent; hsCRP, high-sensitivity C-reactive protein; IL-6, Interleukin-6

^a^ Unadjusted

^b^ Adjusted for age, sex, race, body mass index, etiology of end stage renal disease, diabetes status, transferrin saturation (serum iron/total iron binding capacity), Kt/V, serum parathyroid hormone, and study visit

^c^ Adjusted for Model 2 covariates plus isofurans

^d^ P-values obtained from comparing regression coefficients to coefficient for lowest quartile of each inflammatory marker

**Figure S1: Correlation of baseline inflammatory and oxidative stress markers in maintenance hemodialysis patients**. Panel (A): hsCRP and IL-6; Panel (B): F2-isoprostanes and isofurans
